# Effects of Purple-Fleshed Sweet Potato Lyophilized Powder on the Physicochemical Properties, Lactic Acid Bacteria Viability, Microstructure, and Textural Properties of Stirred Yogurt

**DOI:** 10.3390/foods14020257

**Published:** 2025-01-15

**Authors:** Paulo Cezar da Cunha Júnior, Carlos Alberto Cruz Pinto, Jorge Manuel Alexandre Saraiva, Elisa Helena da Rocha Ferreira

**Affiliations:** 1 Programa de Pós-Graduação em Ciência e Tecnologia de Alimentos, Instituto de Tecnologia, Universidade Federal Rural do Rio de Janeiro (UFRRJ), Seropédica, Rio de Janeiro 23890-000, Brazil; pc.cunha.alim@gmail.com; 2 Centro Federal de Educação Tecnológica Celso Suckow da Fonseca (CEFET/RJ), Valença, Rio de Janeiro 27600-000, Brazil; 3 LAQV-REQUIMTE, Departamento de Química, Universidade de Aveiro, 3810-193 Aveiro, Portugal; carlospinto@ua.pt; 4 Departamento de Tecnologia de Alimentos, Instituto de Tecnologia, Universidade Federal Rural do Rio de Janeiro (UFRRJ), Seropédica, Rio de Janeiro 23890-000, Brazil

**Keywords:** anthocyanins, natural food agent, technological properties, fermented milks

## Abstract

This study proposes the use of lyophilized powder of purple-fleshed sweet potato (LP) as a new multifunctional ingredient to improve the identity and quality parameters of stirred yogurts. The physical and chemical properties, color, monomeric anthocyanin content, lactic acid bacteria viability, water retention capacity, microstructure, and texture were evaluated for yogurts enriched with LP at the levels of 2% (YLP2), 4% (YLP4), and 6% (YPL6), stored for 30 days under refrigeration (4 °C). The results indicated that LP provided different intensities and shades of pink coloration to yogurt, in addition to increasing (*p* < 0.05) the water retention capacity and reducing the water activity. No post-acidification processes were observed during storage. YLP2, YLP4, and YLP6 showed higher stability regarding the number of viable lactic acid bacteria cells compared to the control sample (without enrichment) during storage. Interstitially, adding LP improved the microstructures of the yogurts, promoting more cross-linked networks, with greater uniformity and smaller empty zones, regardless of the level used; in addition, the yogurts (YLP4 and YLP6) were firmer and creamier. These findings demonstrate that LP can be used as a multifunctional ingredient to promote technological/functional improvements, being underscored as a promising natural colorant, stabilizer, emulsifier, and thickener for yogurts.

## 1. Introduction

Yogurt is one of the most economically important dairy products worldwide, with outstanding nutritional quality due to its lipid, protein, mineral, and vitamin content. It results from the fermentation of milk by *Streptococcus thermophilus* and *Lactobacillus delbrueckii* subsp. *bulgaricus* but may also have other microorganisms in its composition [1,2]. These lactic acid bacteria (LAB) have a population density greater than 7 log CFU/g and can bring benefits to human health, such as disease prevention and the strengthening of the immune system, when consumed in adequate quantities and frequencies. Fermentation occurs up to pH values between 4.60 and 4.50, giving rise to a coagulated, acidic, and semi-solid food [3,4].

Synthetic additives can be used to correct technological flaws or improve the technological and sensory characteristics of yogurts, including stabilizers, thickeners, emulsifiers, and colorings, among others [5]. However, these ingredients may present deleterious effects on human health [6], causing consumers to look for more natural options. Thus, their minimal use and/or non-use is required, resulting in changes in the market and products offered, which is mainly represented by the clean label movement [7]. This current trend has stimulated scientific research aiming at the search for alternative ingredients to synthetic additives.

Wang et al. [8] observed increased firmness and cohesiveness of yogurts when enriching them with freeze-dried apple pomace, which reinforced the casein gels. Chen et al. [1] described more homogeneous structures in yogurts incorporated with soy fiber from okara, in addition to observing stronger gels and greater water retention capacity. Du et al. [9] reported increased viscosity, consistency, and firmness of yogurts enriched with blackberry pomace. Improvements in texture, consistency, and cohesiveness were achieved by enriching yogurts with pineapple pomace, indicating a physical structure reinforcement [10]. Therefore, these studies demonstrate that there are diverse possibilities for improvements in the physical, chemical, sensory, and nutritional characteristics of yogurts, especially by using different matrices of vegetable origin, among which the purple-fleshed sweet potato stands out.

Purple-fleshed sweet potato (PFSP) is a root with high levels of bioactive compounds and easy adaptation to different climates and soils, with a high yield per hectare [11]. It has outstanding nutritional quality, showing considerable levels of fiber, vitamins, and minerals and low lipid content [12]. In addition, PFSP is a source of total starch (levels above 50%), an important ingredient for the food industry as it presents numerous technological functions, serving as a thickener, emulsifier, coating and encapsulating material, binding agent, stabilizer, and gelling agent [13]. Among the bioactive compounds from PFSP, anthocyanins stand out. They are natural pigments with recognized antioxidant capacities and have the property of color interconversion, conferring a wide range of colorations with different shades and intensities, which vary according to the pH characteristics of the medium [14]. It is worth noting that studies indicate the beneficial biological effects of anthocyanins, especially those contained in PFSP. These compounds may present antihyperglycemic effects due to the inhibition of the enzyme α-glucosidase [15], as well as antiatherosclerotic [16], antihypertensive [17], anti-inflammatory, and anticancer activity [18] and potential against hepatic inflammation [19]. Thus, it is expected that the incorporation of PFSP would exert several effects on the characteristics of yogurt, mainly due to the functionalities of anthocyanins and starch.

In this context, this study aimed to investigate the use of PFSP in the form of lyophilized powder as a multifunctional ingredient, considering its impact on stirred yogurts’ properties.

## 2. Materials and Methods

### 2.1. Materials and Chemical Reagents

The samples of the purple-fleshed sweet potato (PFSP) were obtained from a family production unit located in Seropédica, Rio de Janeiro, Brazil (22°47′03.6″ south latitude and 43°39′40.1″ west longitude). Pasteurized whole milk (3.6% fat; Vigor, Porto, Portugal) was obtained in a local market (Aveiro, Portugal). Lyofast Y 438 A, a lyophilized culture of *Streptococcus thermophilus* and *Lactobacillus delbrueckii* subsp. *bulgaricus*, was supplied by Sacco Brazil (São Paulo, Brazil).

Sodium hypochlorite was purchased from Ypê (Amparo, São Paulo, Brazil). Sodium hydroxide was purchased from VWR (Leuven, Belgium). Phenolphthalein was purchased from Neon (Suzano, São Paulo, Brazil). Ethanol was purchased from Carlo ERBA Reagents (Val de Reuil, France). Sodium acetate anhydrous was purchased from Labkem (Barcelona, Spain). Potassium chloride and Rhodamine B were purchased from Sigma-Aldrich (Seelze, Germany). MRS (De Man, Rogosa and Sharpe) agar was purchased from Himedia (Maharashtra, India).

The lyophilized powder (LP) of PFSP was obtained at the Instrumental Laboratory of the Graduate Program in Food Science and Technology of the Federal Rural University of Rio de Janeiro (Seropédica, Rio de Janeiro, Brazil), while other experimental practices were carried out at the Department of Chemistry of the University of Aveiro (Aveiro—Portugal).

### 2.2. Preparation of Purple-Fleshed Sweet Potato Lyophilized Powder (LP)

The PFSP were manually harvested, selected, washed, and sanitized by immersion in a sodium hypochlorite solution (200 ppm for 15 min). Then, they were peeled and chopped into 5 cm^2^ pieces, following the methodology proposed by Cunha Júnior et al. [20].

The heating treatment was carried out by immersing the samples in water at 80 °C for 10 min (1:3 *w*/*w* ratio). After the heat treatment, the potatoes and cooking water were processed in an industrial blender (model LI-1,5-N, Skymsen, Santa Catarina, Brazil) for 10 min. The puree obtained was placed in an airtight plastic bag and frozen at −18 °C in a conventional freezer for 7 days. Subsequently, the puree was lyophilized in a benchtop freeze dryer (Liotop^®^, model L101, São Paulo, Brazil) for an average period of 5 days, crushed in an industrial blender, and placed in a 710 μm granulometric analysis sieve. This process resulted in the lyophilized powder of purple-fleshed sweet potato (LP), which was stored in airtight containers under refrigeration (4 °C).

### 2.3. Production of LP-Enriched Stirred Yogurt

Pasteurized whole milk was heated to 43 °C, and lyophilized cultures of *Streptococcus thermophilus* and *Lactobacillus delbrueckii* subsp. *bulgaricus* were added in the proportion of 0.07 g L^−1^ of milk, following the manufacturer’s recommendation. It was then incubated in a water bath at 43 °C until it reached a pH value between 4.5 and 4.6. The pH was determined using a potentiometer (TitroMatic pH-Stat 1S, Crison, Barcelona, Spain). After coagulation, the yogurt was divided into four equal parts. Three parts were enriched with LP using three ratios (2% *w*/*w*—YLP2, 4% *w*/*w*—YLP4, and 6% *w*/*w*—YLP6). Thus, four different samples were obtained: control (without addition of LP), YLP2, YLP4, and YLP6. The samples were fractionated in airtight plastic containers containing 30 g of yogurt each and stored under refrigeration (4 ± 1 °C).

### 2.4. pH and Titratable Acidity (TA)

The pH and titratable acidity were determined using an automatic titrator with an attached potentiometer (TitroMatic pH-Stat 1S, Crison, Barcelona, Spain) at 0, 10, 20, and 30 days of storage under refrigeration at 4 °C. Titratable acidity (TA) was determined using 0.1 M sodium hydroxide solution as a titrant and 1% phenolphthalein ethanol solution as an indicator. The results were expressed as % lactic acid.

### 2.5. Total Monomeric Anthocyanin Content (TMA) and Instrumental Color Analysis

The total monomeric anthocyanin (TMA) values of the LP and yogurt samples were determined using the procedure reported by Du et al. [9], with modifications. The extracts were prepared with 1.5 g of the samples and 30 mL of an acidified ethanolic aqueous solution (60% *v*/*v*) in an ultrasound bath for 2 h. The samples were centrifuged at 3570× *g* for 20 min at 4 °C. The supernatants were filtered, and the TMA was measured by the differential pH method (pH 1.00 and pH 4.50) described by Lee et al. [21].

The extract (2.00 mL) was added to 3.00 mL of potassium chloride buffer (0.025 M/pH 1.00) and 3.00 mL of sodium acetate buffer solution (0.4 M/pH 4.00). The mixture was homogenized and allowed to rest for 15 min. Next, the absorbance was measured at 530 (A_530 nm/pH 1.00_ and A_530 nm/pH 4.50_) and 700 nm (A_700 nm/pH 1.00_ and A_700 nm/pH 4.50_) using a spectrophotometer (Multiskan GO, Thermo Scientific, Vantaa, Finland). The TMA was quantified according to Equations (1) and (2), and the result was expressed as μg of cyanidin 3-glycoside g^−1^ of sample.

A = [(A_530 nm/pH 1.00_ − A_700 nm/pH 1.00_) − (A_530 nm/pH 4.50_ − A_700 nm/pH 4.50_) (1)

(2) $$TMA=\left(\frac{A}{\varepsilon \times L}\right)\times MM\times 100\times f_{d}$$

where A is the absorbance, corrected by the difference between the absorbances recorded in the readings at pH 1.00 and pH 4.50; ε is the molar extinction coefficient of anthocyanins; *L* is the length of the optical path (1 cm); *MM* is the molecular weight of anthocyanins; $f_{d}\;$ is the dilution factor of the sample; TMA is the total anthocyanin content.

Color was measured using a Konica Minolta Colorimeter cm-2600d (Konica Minolta^®^, Osaka, Japan). The samples were placed on glass plates and the CIELab parameters L*, a*, and b* were determined, where the value of L* ranges from 0 (black) to 100 (white), the coordinate a* represents red (positive) to green (negative), and b* represents yellow (positive) to blue (negative). The evaluations of the yogurt color parameters were performed at 0, 10, 20, and 30 days of storage under refrigeration at 4 °C.

### 2.6. Total Viable Lactic Acid Bacteria (LAB) Count

Total counts of viable lactic acid bacteria (LAB) were performed at the intervals of 0, 10, 20, and 30 days of storage under refrigeration (4 °C), using the microdroplet technique [22]. From serial dilutions, 20 μL droplets were incubated on the surfaces of plates containing De Man, Rogosa, and Sharpe (MRS) agar. The plates were incubated at 37 ± 1 °C for 72 h in an unventilated oven under anaerobic conditions. The result was expressed as the logarithm of the number of colony-forming units per g (log CFU g^−1^).

### 2.7. Water Holding Capacity (WHC) and Water Activity (WA)

The water holding capacity (WHC) was measured using the procedure described by Du et al. [9], with modifications. The samples (10 g) were centrifuged for 10 min at 3570× *g*. Then, the expelled water was removed and weighed. The WHC was expressed as % and calculated according to Equation (3):

(3) $$WHC\left(\%\right)=\left(\frac{\mathrm{sample}\;\mathrm{weight}-\mathrm{weight}\;\mathrm{of}\;\mathrm{expelled}\;\mathrm{whey}}{\mathrm{sample}\;\mathrm{weight}}\right)\times 100$$

The WA of the yogurt samples was determined using a digital hygrometer (Humimeter RH2, Schaller Messtechnik GmbH, St. Ruprecht an der Raab, Austria). The yogurt samples were left to rest until they reached a temperature of 4 °C. For the equipment, the rest period was 30 min, to adjust to room temperature. Then, 200 g of yogurt was transferred to the water activity analysis chamber (screw-top jar). The system was left at rest for the measurement of WA at 4 °C and the reading was performed when the 3 decimal places were unchanged.

### 2.8. Laser Scanning Confocal Microscopy

The microstructures of the yogurts were studied at 0 days using laser scanning confocal microscopy, following the methodology proposed by Körzendörfer et al. [23], with minor modifications. Yogurt samples (500 μL) were stained with 10 μL of an aqueous solution of Rhodamine B (0.2%) and stored at 4 °C for 1 h. The stained samples were analyzed under a fluorescence microscope, the Imager M.2 Widefield (Zeiss, Jena, Germany). Three-dimensional images were obtained by scanning the samples in a defined slice along the *z*-axis.

### 2.9. Texture Analysis

The texture analysis of the enriched yogurt samples was performed in a TA-XT2i texture analyzer (Stable Micro Systems, Godalming, Surrey, UK). Yogurts (30 g) were transferred to plastic pots and left to rest under refrigeration (4 °C) for 24 h. The texture profile analysis was carried out using a circular probe (40 mm diameter × 5.7 mm thickness) and a load cell (5 kg). The analysis conditions were as follows: pre-test speed—1.0 mm/s; test speed—1.7 mm/s; post-test speed—1.7 mm/s; 2 penetration cycles; distance traveled by the device on the specimen—2 mm; contact force—0.049 N; contact time—5 s. The parameters obtained were the firmness, gumminess, and chewiness.

### 2.10. Statistical Analysis

All experiments were performed in triplicate. The results were expressed as the mean ± standard deviation. The data were subjected to an analysis of variance (ANOVA). The means were compared by the Tukey test and the significance level was set at 5% (*p* < 0.05). The statistical analysis was performed using Statistic 10.0 (Statsoft^®^, Tulsa, OK, USA).

## 3. Results and Discussion

### 3.1. pH and Titratable Acidity (TA)

pH and acidity are important parameters regarding the safety and technological aspects during yogurt processing, in addition to having an intrinsic and direct relationship with the sensory characteristics of this product. Thus, the maintenance of the pH and acidity throughout storage is a crucial requirement for a product within the identity and quality parameters expected for yogurt [24]. The enrichment of yogurt with LP did not promote significant changes (*p* < 0.05) in the pH value at day 0 (Table 1). However, reductions in pH were observed during storage, being more evident for the control sample, since it showed a reduction of 0.23, while the enriched samples (YLP2, YLP4, and YLP6) had variations between 0.08 and 0.12. The samples showed pH values in the range of 4.17 to 4.44, providing a natural barrier that contributed to maintaining the microbiological quality of the product, as pH values below 4.50 can inhibit the development of most pathogenic and spoilage microorganisms [24].

As expected, the titratable acidity variation, expressed as the lactic acid concentration, complemented the results of the pH analyses. Significant increases (*p* < 0.05) were observed over the 30 days of storage (Table 1), which were more pronounced in the control and YLP2 samples. This is in line with the fact that these samples were the ones with the greatest variations in pH when comparing 0 and 30 days of storage, while the YLP4 sample did not have a significant variation in its lactic acid content (*p* < 0.05) over the 30 days of storage, and YLP6 varied only between day 0 and the other storage times (Table 1).

These findings indicate that there was no evident post-fermentation acidification process, suggesting that the production of metabolites by the microorganisms occurred discreetly during storage. This scenario is highly promising, since post-fermentation acidification is considered a technological defect for yogurts, leading to the product’s rejection by consumers and reducing its shelf life [25,26].

### 3.2. Total Monomeric Anthocyanin Content (TMA) and Instrumental Color Analysis

Table 2 shows the anthocyanin content and color parameters (L*, a*, and b*) obtained from the samples during storage under refrigeration at 4 °C.

The enriched samples (YLP2, YLP4, and YLP6) differed significantly (*p* < 0.05) from the control treatment for all parameters analyzed in the instrumental color assay (L*, a*, and b*). In addition, significant differences (*p* < 0.05) in anthocyanin content were observed among the enriched samples. Color change during storage is expected both in dairy breeders and in foods containing anthocyanins, since lipids and phenolic compounds can undergo oxidation processes, resulting in the formation of dark-colored compounds. Moreover, pigments, like anthocyanins, may also degrade, promoting color changes throughout storage [27,28].

Regarding the L* parameter, reductions were observed in an inverse way regarding the enrichment, i.e., the higher the level of LP, the lower the luminosity of the samples, indicating that they became darker after the addition of LP (Table 2). However, YLP2, YLP4, and YLP6 showed some stability in relation to L* over the 30 days of storage as the luminosity did not have significant variations (*p* < 0.05). In the control sample, the L* parameter increased at 10 days of storage and decreased at 20 days (Table 2). Similar results have been reported in the scientific literature. Gavril et al. [29] studied the enrichment of yogurt with pumpkin peel power. The authors observed an increase in the L* parameter at 7 days of storage in the control sample (without pumpkin peel power). Gamage et al. [30] enriched yogurt with blue pea flower. When evaluating the total color variation throughout storage, the authors observed greater variation at 18 days than at 24 days of storage (reference time: 0 days of storage). The observations and findings of the present study reveal that the variation in the luminosity of yogurt throughout storage is recurrent and expected. These changes can be justified by several factors, such as the composition and physicochemical properties of the milk/yogurt, as well as the reduction in pH during storage, which can promote changes in the parameter L* [31].

As shown in Table 2, YLP2, YLP4, and YLP6 presented pink coloration in different shades, corroborating the readings observed for a*. YLP2, YLP4, and YLP6 had positive values, ranging from 7.00 to 11.71, while the control sample showed negative values, between −0.95 and −1.46, indicating the presence of red stains (positive values for a*) in the enriched samples. Significant reductions (*p* < 0.05) in the a* readings for YLP2, YLP4, and YLP6 were determined over refrigerated storage. However, although significant, these reductions were not of great magnitude (Table 2).

The coloration observed can be justified by the color interconversion property of anthocyanins, which may modify their chemical structures according to the pH characteristics of the medium, conferring a wide range of colorations with different shades and intensities. At pH values close to 4.50 (Table 1), in general, anthocyanins tend to have a pink–lilac color [14]. As expected, there was a gradual increase in the anthocyanin content that was proportional to the increase in the level of LP, with values in the range of 64.47 and 96.44 μg cyanidin-3-glucoside g^−1^ of yogurt, which had a direct correlation with the significant increases (*p* < 0.05) for parameter a*, compared to the control treatment.

Similarly, parameter b* was also consistent with the anthocyanin content. The control treatment showed positive values, indicating that its color tended towards yellow (Table 2). In contrast, YLP2, YLP4, and YLP6 showed negative values for this parameter, suggesting stains tending towards blue, which, in association with parameter a*, can induce slightly purple stains. For the control treatment, parameter b* was stable throughout storage, with significant changes (*p* < 0.05) only at day 10. However, the enriched samples were unstable, with statistically significant increases (*p* < 0.05) in the b* values over the 30 days of storage (Table 2).

The anthocyanin content found in the present study was higher than that reported by other authors who supplemented yogurts with matrices and extracts containing anthocyanins. Machado et al. [32] determined content varying from 1.03 to 5.87 μg cyanidin-3-glycoside g^−1^ in yogurts enriched with jabuticaba bagasse, while Anuyahong et al. [25] obtained content between 28.8 and 93.8 μg cyanidin-3-glycoside g^−1^ when adding red Thai rice extracts to stirred yogurt and 79.09 μg cyanidin-3-glycoside g^−1^ at 0 days of storage in yogurts with blackberry pomace. Notably, in this study, the authors reported an increase in the concentration of anthocyanins after 28 days of storage, registering levels of 131.43 and 309.67 μg cyanidin-3-glycoside g^−1^ for samples containing 1% and 3% of blackberry pomace, respectively. Therefore, increments with such magnitude have not been reported in the literature. Jaster et al. [33] enriched yogurts with strawberry pulp, a recognized source of anthocyanins, and obtained content ranging from 18.60 to 29.90 μg cyanidin-3-glucoside g^−1^.

In addition to increasing the content of anthocyanins and providing color to yogurts, other technological aspects of anthocyanins from PFSP already described in the scientific community can further stimulate the use of LP as a food ingredient. In a study conducted by Tsukui et al. [34], the anthocyanins present in PFSP were more stable in the face of heating and UV light irradiation than those from other fruits and vegetables, such as apples, cabbage, and strawberries, due to the high degree of acylation. Moreover, according to Oliveira et al. [35], anthocyanins from PFSP have greater resistance to gastrointestinal tract conditions when compared to less complex anthocyanins, such as the anthocyanins present in red wine. Therefore, these advantageous characteristics highlight the potential use of PFSP as a source of natural food ingredients.

### 3.3. Total Viable Lactic Acid Bacteria (LAB) Count

**Figure 1:**
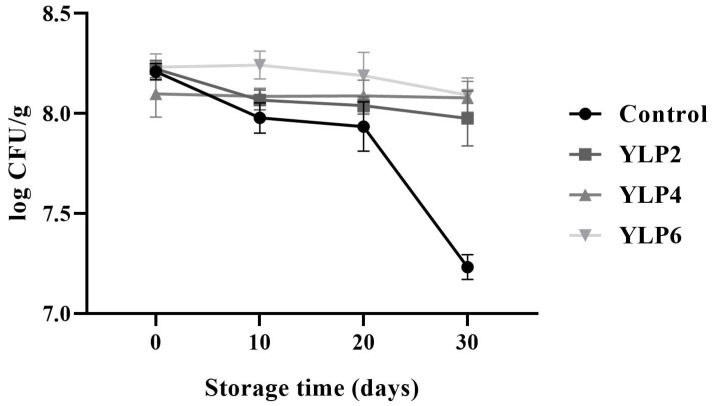
Total count of viable lactic acid bacteria (LAB) of stirred yogurts enriched with lyophilized powder of purple-fleshed sweet potato during 30 days of storage under refrigeration (4 °C).

LP promoted greater stability in the total viability of lactic acid (LAB) bacteria over the 30 days of storage (Figure 1). All samples had counts higher than 7.00 log CFU/g. Therefore, it was possible to observe that the control treatment presented lower counts and more evident variations throughout storage (Figure 1).

The results revealed that the compounds present in LP promoted the maintenance of Streptococcus thermophilus and Lactobacillus bulgaricus. This behavior was also reported by other authors due to the enrichment of dairy products with ingredients from plant matrices. Dantas et al. [36] enriched yogurt with xique-xique (Pilosocereus gounellei) flour (1 and 2%) and determined higher counts of Lactobacillus bulgaricus and Limosilactobacillus mucosae over 28 days of storage, when compared to the control treatment. Almusallam et al. [37] observed reductions in LAB counts from 14 days of storage in yogurts enriched with extracts of date palm (*Phoenix dactylifera* L.) spikelets. Azevedo et al. [38] studied fermented milk containing grape pomace extracts, determining higher counts in the supplemented samples. Moreover, it is worth noting that the latter study also used a matrix rich in anthocyanins.

### 3.4. Water Holding Capacity (WHC) and Water Activity (WA)

Syneresis is one of the main technological defects in yogurts and is characterized by the expulsion of whey by the clot. Its foundation lies in the balance between the forces of attraction and repulsion in the protein network, especially by casein, formed during yogurt fermentation. This aspect is an important quality parameter, which is directly related to product acceptance [25]. Syneresis may be measured through the physical quantification of the water retention capacity since yogurts with higher water retention capacity have lower syneresis.

YLP2, YLP4, and YLP6 statistically differed (*p* < 0.05) from the control treatment during storage (30 days), where the water holding capacity (WHC) increased (*p* < 0.05) after enriching the yogurts with LP (Table 3), resulting in less syneresis.

In addition, the higher the level of enrichment, the higher the WHC, indicating that LP promoted technological improvements in the yogurts. It was more pronounced in the YLP4 and YLP6 samples, which showed WHC increases close to 15 and 19% at day 0 and 10 and 15% at 30 days of storage, respectively (Table 3). The control treatment did not show significant variations (*p* < 0.05) over storage, while YLP2, YLP4, and YLP6 were stable after day 10.

Adding LP increased the content of total solids. It modified the physical and chemical structures of the yogurts, which presented higher water holding capacity. These findings agree with other studies, like the one by Chen et al. [1], who reported that adding okara fiber to yogurts increased their water holding capacity by 80% in supplementation at 0.75, 1.00, and 2.00%. In yogurts enriched with red rice extracts, Anuyahong et al. [25] observed a slight reduction in syneresis. Du et al. [9] also described lower whey loss over storage in yogurts enriched with pomace mulberry (a maximum value of 44%); however, it was less expressive than in the present study, where values of WHC higher than 69% were found.

The increased WHC of the enriched samples may be mainly attributed to the starch present in LP. Starch is a carbohydrate with several technological functions for the food industry, including emulsifying, stabilizing, and thickening capacities [13]. In addition, as starch has great water retention capacity, it may promote stronger interactions between milk proteins, making the network firmer, less cross-linked, and more structured [39]. Moreover, the polyphenols present in LP can form soluble complexes with the casein molecule, increasing hydrophobic interactions with the association of hydrogen bonds and, thus, minimizing syneresis [25].

The water activity (WA) of the yogurt samples showed values from 0.885 to 0.927. All samples differed statistically from each other (*p* < 0.05). As expected, the water activity readings were inversely proportional to the enrichment levels (Table 3). Adding LP to the yogurts caused an increase in the total solid content, reducing the WA due to the interactions formed between the LP components and the water present in the yogurts. In this way, starch plays a crucial role as it has a great capacity to expand and retain water [39,40].

Such results are of great importance regarding microbiological stability. The lower WA observed with the addition of LP, associated with other characteristics, such as refrigerated storage, pH, and acidity, can increase yogurts’ shelf life. According to Jay [41], a reduction in WA can increase the growth lag phase, making it possible to reduce the growth rate and the density of the final population. The author also states that a reduction in the WA can also impact the metabolism of pathogenic and spoilage microorganisms, since, at the cellular level, all chemical reactions require an aqueous environment.

### 3.5. Laser Scanning Confocal Microscopy

**Figure 2:**
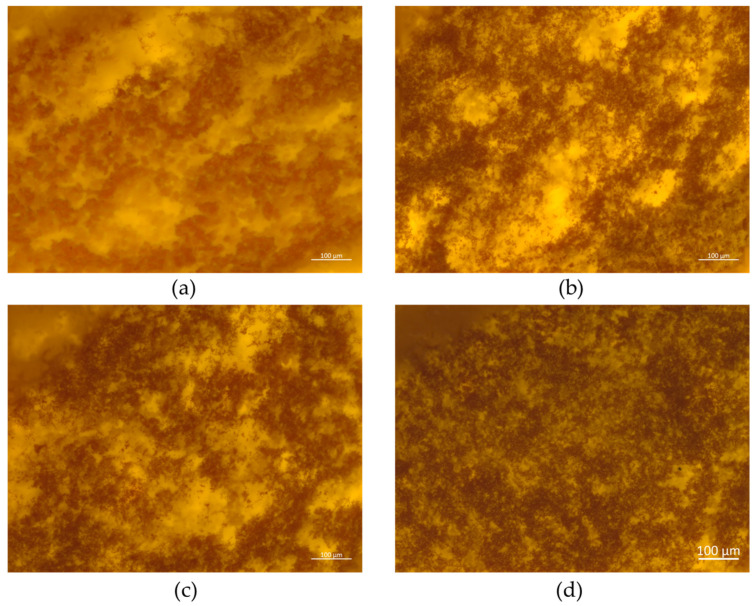
Laser scanning confocal photomicrographs of stirred yogurts enriched with lyophilized purple-fleshed sweet potato powder (LP): (**a**) control, (**b**) YLP2, (**c**) YLP4, and (**d**) YLP6 at 0 days of storage.

Photomicrographs indicated that all yogurt samples showed a relatively uniform and porous lattice structure, with visual differences between the structures of the different treatments (Figure 2). The addition of LP significantly reduced the porous spaces of the samples, resulting in a denser yogurt with a more uniform and organized microstructure. Moreover, it also promoted greater protein aggregation (yellowish zones), which was increasingly evident as the level of LP increased.

These results corroborate the other properties evaluated in the present study (water holding capacity, water activity, and texture). From the photomicrographs, it was possible to visualize the improvement in the protein network, with an increase in cross-linking proportional to the level of LP. Thus, the higher the LP level, the greater the protein gel network’s cross-linking, which was evidenced by the increase in the water retention capacity and the firmer and creamier yogurts, as well as by the reduced available water.

According to the scientific literature, more cross-linked structures and uniform networks can be achieved by adding blackberry pomace (1–3%) to yogurts [9]. Wu et al. [42] also observed that yogurt without any type of enrichment was composed of protein aggregates with empty/free zones, revealing that supplementing yogurts with rice bran resulted in a more compact network with reduced free zones. Such changes in the microstructure may lead to less rearrangement during storage, reducing syneresis. The same was noticed in the present study, since the samples enriched with LP showed higher water capacity when compared to the control treatment (Table 3), evidencing the important technological role of starch in yogurt processing, since rice bran and PFSP have starch in their compositions. Thus, it was found that LP increased the stability and uniformity of the yogurts, minimizing possible technological failures in the final product.

### 3.6. Texture Analysis

**Figure 3:**
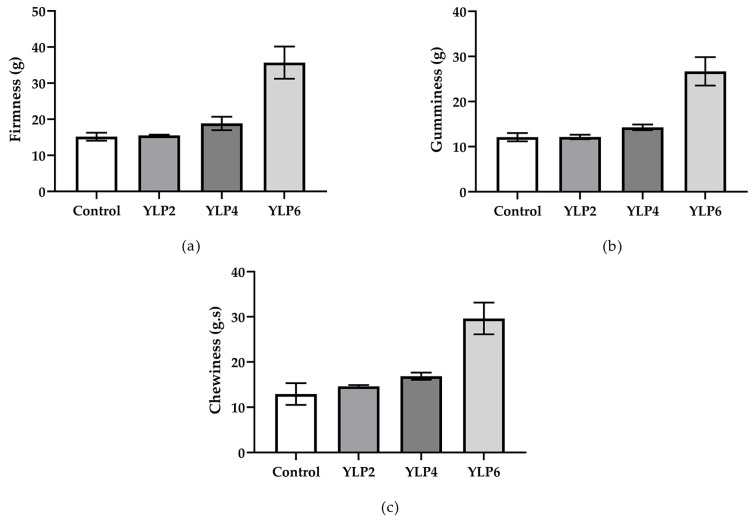
Texture profiles of stirred yogurts enriched with lyophilized purple-fleshed sweet potato powder (LP) from PFSP at concentrations of 0%, 2%, and 4% at 0 days of storage: (**a**) firmness, (**b**) gumminess, and (**c**) chewiness.

The LP significantly changed (*p* < 0.05) the texture profiles of the yogurts when added at 4 and 6% (YPL4 and YLP6), increasing their firmness, gumminess, and chewiness (Figure 3).

According to Anuyahong et al. [25], texture is an important quality parameter for yogurt. The results obtained indicate that YLP4 and YLP6 were firmer and had higher viscosity and creaminess. Such changes were due to the increase in total solids and the formation of new interactions provided by the addition of LP, especially due to the presence of starch. As reported by He et al. [40], starch can improve yogurt’s texture, mainly by increasing its viscosity. According to Jia et al. [39], the production of firmer yogurts with the addition of starchy sources is due to the expansion property of starch, which can play an important technological role in yogurt’s texture.

In general, enrichment with matrices containing polysaccharides promotes positive changes in the texture profiles of yogurts. Wang et al. [8] obtained firmer and more consistent yogurts after the addition of apple pomace. Similar findings were described by Cota-López et al. [43], who evaluated the effect of adding retrograde starches with different levels of amylose in Greek yogurt, and Meena et al. [10], who studied yogurts with pineapple pomace added at minimal levels (0.1, 0.25, and 0.5%). Therefore, the improvement in the texture profile of yogurt by enrichment with LP is highly promising, since firmer and creamier yogurts can be produced without using synthetic food additives, such as thickeners and emulsifiers, meeting the current market and consumption trends related to the clean label movement [7].

## 4. Conclusions

The overall results demonstrate the great potential of LP to improve the technological properties of stirred yogurt. The addition of LP resulted in pink colors of different shades, with low variation throughout storage. Moreover, it also increased the water retention capacity and reduced the water activity of the samples. Samples containing 4 and 6% LP showed greater firmness and creaminess, and post-acidification was not observed during storage in any of the treatments; these aspects are of great interest for the maintenance of yogurt’s quality. The LP provides stable and structured yogurts, with more cross-linked, compact networks and fewer empty areas. Moreover, higher bioactivity may be expected due to the presence of anthocyanins from LP, and this should be studied further.

## Figures and Tables

**Table 1:** Variations in pH and titratable acidity of stirred yogurt enriched with lyophilized powder of purple-fleshed sweet potato (LP) during 30 days of storage under refrigeration (4 °C).

Storage Time (Days)	pH				Titratable Acidity (Lactic Acid %)			
	Control	YLP2	YLP4	YLP6	Control	YLP2	YLP4	YLP6
0	4.40 ^Aa^ ± 0.02	4.40 ^Aa^ ± 0.02	4.42 ^Aa^ ± 0.01	4.44 ^Aa^ ± 0.02	0.76 ^Aa^ ± 0.05	0.79 ^Ba^ ± 0.02	0.81 ^Aa^ ± 0.03	0.80 ^Ba^ ± 0.01
10	4.40 ^Aa^ ± 0.02	4.37 ^ABa^ ± 0.01	4.37 ^Ba^ ± 0.01	4.44 ^ABa^ ± 0.05	0.82 ^Aa^ ± 0.04	0.79 ^Ba^ ± 0.06	0.83 ^Aa^ ± 0.03	0.83 ^ABa^ ± 0.05
20	4.32 ^Bc^ ± 0.01	4.34 ^Bbc^ ± 0.03	4.37 ^Bb^ ± 0.00	4.40 ^Ba^ ± 0.01	0.80 ^Ab^ ± 0.06	0.93 ^Aa^ ± 0.01	0.86 ^Aab^ ± 0.06	0.84 ^ABb^ ± 0.03
30	4.17 ^Cc^ ± 0.02	4.28 ^Cb^ ± 0.03	4.33 ^Ca^ ± 0.02	4.36 ^Ba^ ± 0.02	0.93 ^Aa^ ± 0.16	0.96 ^Aa^ ± 0.04	0.88 ^Ab^ ± 0.02	0.88 ^Ab^ ± 0.01

Values in the same column for the same parameter with different superscript uppercase letters indicate significant difference (*p* < 0.05); Values in the same row for the same parameter with different superscript lowercase letters indicate significant difference (*p* < 0.05).

**Table 2:** Total monomeric anthocyanin content (TMA) and instrumental color parameters (L*, a* and b*) of stirred yogurts enriched with lyophilized powder of purple-fleshed sweet potato (LP) during 30 days of storage under refrigeration (4 °C).

Treatment *		TMA **	Storage Time (Days)	L*	a*	b*
Control	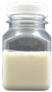	n/a ***	0	73.43 ^Ba^ ± 0.75	−1.01 ^Ac^ ± 0.03	6.10 ^Ba^ ± 0.04
			10	81.86 ^Aa^ ± 1.85	−1.46 ^Bd^ ± 0.00	6.72 ^Aa^ ± 0.19
			20	72.58 ^Ba^ ± 2.66	−0.96 ^Ac^ ± 0.11	6.01 ^Ba^ ± 0.26
			30	75.20 ^ABa^ ± 5.07	−1.11 ^Ac^ ± 0.11	6.37 ^ABa^ ± 0.23
YLP2	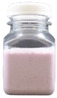	64.47 ^C^ ± 0.91	0	60.97 ^Bb^ ± 1.49	8.33 ^Ab^ ± 0.26	−2.07 ^Db^ ± 0.24
			10	64.35 ^Ab^ ± 2.35	7.83 ^ABc^ ± 0.30	−1.02 ^Cb^ ± 0.15
			20	64.85 ^Ab^ ± 2.73	7.34 ^Bb^ ± 0.36	−0.50 ^Bb^ ± 0.11
			30	66.58 ^Ab^ ± 2.88	7.04 ^Bb^ ± 0.32	−0.16 ^Ab^ ± 0.03
YLP4	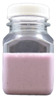	83.58 ^B^ ± 0.85	0	60.30 ^Ab^ ± 3.12	11.39 ^Aa^ ± 0.91	−4.18 ^Dc^ ± 0.51
			10	61.55 ^Abc^ ± 0.59	10.04 ^ABb^ ± 0.19	−2.60 ^Cc^ ± 0.06
			20	60.95 ^Ab^ ± 2.40	9.30 ^BCa^ ± 0.46	−1.79 ^Bc^ ± 0.29
			30	62.44 ^Ab^ ± 2.23	8.84 ^Ca^ ± 0.42	−1.08 ^Ac^ ± 0.12
YLP6	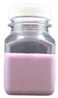	96.44 ^A^ ± 2.75	0	55.68 ^Bc^ ± 0.79	11.71 ^Aa^ ± 0.30	−4.63 ^Dc^ ± 0.24
			10	59.27 ^Ac^ ± 1.08	10.85 ^ABa^ ± 0.27	−3.24 ^Cd^ ± 0.22
			20	57.80 ^ABc^ ± 0.92	10.20 ^BCa^ ± 0.27	−2.54 ^Bd^ ± 0.21
			30	57.01 ^ABc^ ± 1.24	9.25 ^Ca^ ± 0.36	−1.33 ^Ad^ ± 0.06

Values in the same column and the same treatment with different superscript uppercase letters indicate significant difference (*p* < 0.05); Values in the same column and for the same storage time with different superscript lowercase letters indicate significant difference (*p* < 0.05); * The images refer to yogurts at 0 days; ** TMA unit: μg cyanidin-3-glycoside g^−1^ yogurt; *** n/a: not applicable.

**Table 3:** Water holding capacity (WHC) and water activity (WA) of stirred yogurts enriched with lyophilized powder of purple-fleshed sweet potato (LP).

Parameter	Storage Time (Days)	Control	YLP2	YLP4	YLP6
WHC (%)	0	49.13 ^Da^ ± 3.22	52.93 ^Ca^ ± 0.17	65.50 ^Ba^ ± 0.06	69.80 ^Aa^ ± 1.38
	10	48.21 ^Da^ ± 0.34	51.18 ^Ca^± 1.04	57.12 ^Bb^ ± 1.35	61.36 ^Ab^ ± 0.74
	20	47.76 ^Da^ ± 1.36	50.66 ^Cb^ ± 0.37	56.16 ^Bb^ ± 2.16	60.49 ^Ab^ ± 1.39
	30	45.51 ^Da^ ± 1.37	49.88 ^Cab^ ± 2.63	54.97 ^Ab^ ± 0.57	60.95 ^Ab^ ± 6.70
WA *	0	0.927 ^A^ ± 0.001	0.919 ^B^ ± 0.002	0.909 ^C^ ± 0.001	0.885 ^D^ ± 0.005

Values in the same row with different superscript uppercase letters indicate significant difference (*p* < 0.05); Values in the same column with different superscript lowercase letters indicate significant difference (*p* < 0.05); * assay performed only within 0 days.

## Data Availability

The original contributions presented in this study are included in the article. Further inquiries can be directed to the corresponding authors.
